# Your Data to Explore: An Interview with Anne Wojcicki

**DOI:** 10.1371/journal.pgen.1005548

**Published:** 2015-10-15

**Authors:** Jane Gitschier

**Affiliations:** Departments of Medicine and Pediatrics and Institute for Human Genetics, University of California San Francisco, San Francisco, California, United States of America

How can we not be intrigued by secrets held within our genomes? 23andMe erupted onto the biotech landscape in 2006 to satisfy that curiosity with its promise of genetics fun. For a little bit of spit and a few hundred dollars, we could check out genetic predictions for traits we already know we have—perhaps blonde hair or blue eyes—as well as susceptibilities for diseases we may have yet to develop, such as cancer or diabetes. While other small companies took on sober analysis of disease traits and data delivery back to the physician or patient with genetic counseling, 23andMe eschewed the traditional paternalism of American medicine and espoused direct-to-consumer testing. Added to that moxie was a snappy high-tech pedigree. The company was founded by Anne Wojcicki, Linda Avey, and Paul Cusenza and bankrolled in some measure by Google’s Sergey Brin, with whom Wojcicki has two children but from whom she is now divorced.

I have to admit that I have been rooting for 23andMe since its inception (full disclosure: I have no financial link to this company, but I have used their service). I like its efficiency and its premise that individuals, with today’s technology, should have full access to their own genetic information, despite the caveat of sometimes challenging interpretation. I also like the idea of people contributing directly to human genetics research online, something 23andMe incorporated into their model right from the beginning. With time, they have developed algorithms for finding biological relatives and have now partnered with several pharmaceutical companies to mine medical information contributed directly by their consumers. 23andMe has also suffered some setbacks, most prominently a 2013 cease-and-desist order from the American Food and Drug Administration (FDA) against providing medically relevant information. [NB: Earlier this year, the FDA cleared the company’s Bloom Syndrome Carrier Status report, the first direct-to-consumer genetic test ever approved for marketing by the FDA.]

**Fig 1:**
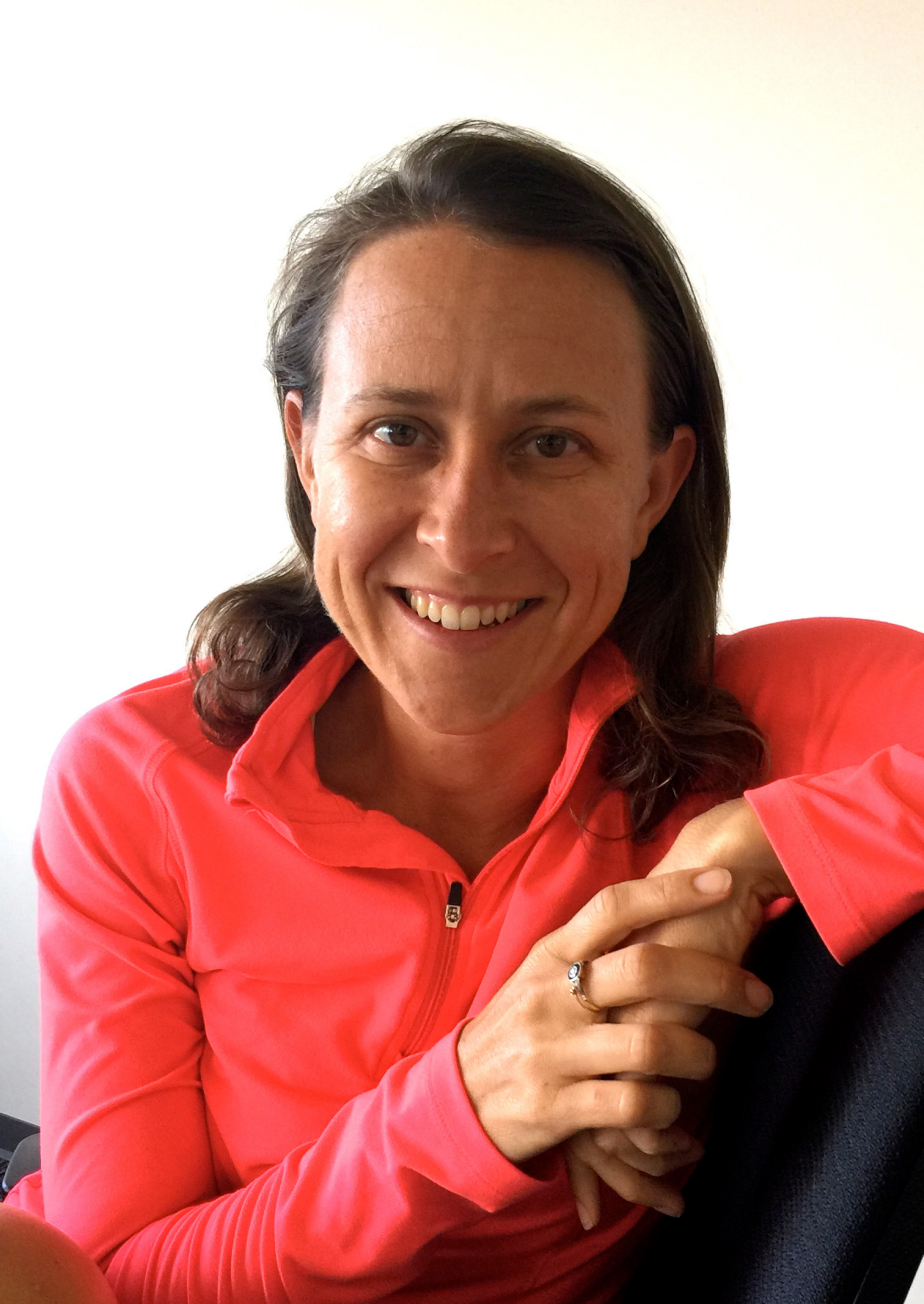
Anne Wojcicki at the 23andMe offices in Mountain View, California, courtesy of 23andme.

I emailed CEO Anne Wojcicki (Fig 1) to request an interview, and she responded, “Yes, I love PLOS,” but securing a time slot proved challenging. We settled on a single hour wedged into the day after 23andMe moved to new quarters in downtown Mountain View, California. It is a beautifully outfitted space with a roof garden, organic lunches and snacks, charge stations for electric cars, and…pet dogs! Clad in her trademark spandex bike shorts and fleece top, Wojcicki demurred on a photo as we settled into a glass cube adjacent to her “open-access” desk, but promised to send one along later.

**Wojcicki**: Do you remember when our first publication [in *PLOS Genetics*] came out?

**Gitschier**: Yes. It was about mapping a few common traits, like asparagus anosmia, photic sneeze reflex, and freckling.

**Wojcicki**: You guys wouldn’t publish it because we didn’t have IRB [Institutional Review Board approval], so we had to go back and get it. What PLOS wrote in the editorial was fascinating. It was good!

**Gitschier**: Well, let’s start with that because it will lead into some other stuff. You didn’t anticipate that IRB approval was going to be an issue, but at the end of the day, it may have forced you to dig a little deeper.

**Wojcicki**: Well, we did think about IRB, and we were told that we are not doing human subjects research. The original IRBs told us that because we’re not really touching the human, we’re not doing an experiment.

**Gitschier**: That’s weird.

**Wojcicki**: No! We get that ruling repeatedly, as we’re launching in different countries.

**Gitschier**: Well, as a human geneticist and a past member of an IRB, I’d say you are definitely doing human subjects research: you’re collecting DNA, you’re taking biometric information of some kind, so that’s—under my definition—human subjects research.

**Wojcicki**: My point is that we actually had thought about it. We had to go back and say, “No, we want to be approved,” and then go back and get that approval.

**Gitschier**: And so the more recent hurdle, of course, is the FDA. Did their position surprise you?

**Wojcicki**:Originally we thought we would not need FDA approval, since most diagnostics in this country are not regulated by the FDA. We had CLIA [Clinical Laboratory Improvement Amendments] approval, and we operated under CLIA, the way most genetic tests do. The FDA then said we are a medical device, and not a diagnostic under CLIA.

**Gitschier**: There were one or two studies in which people sent their spit to several different genetic testing companies and got different interpretations. I got the impression that that was what really precipitated the FDA letter.

**Wojcicki**: I don’t think that is what precipitated it. Interpretation was an issue. But a lot of the FDA comments were focused on, “We want to make sure that the underlying platform is accurate.” So you, being a geneticist, you understand the data quality…

**Gitschier**: …is superb!

**Wojcicki**: Us, Illumina, Navigenics—we are all using the same data, but we were using different studies to interpret it. And I would argue that happens all the time today.

**Gitschier**: Good point. Each doctor might interpret the results differently.

**Wojcicki**: Right. You have physicians or researchers taking different datasets and interpreting. So, something like cystic fibrosis and Δ508: nobody argues what that association is [i.e., a one-to-one correlation between mutation and disease]. But for something like prostate cancer, when you are trying to come up with risk metrics, it’s a question of what studies you actually include in that [analysis]. That was something the press loved to harp about, but it ended up making it more confusing for everyone.

**Gitschier**: So, now you’re choosing to address the FDA challenge, because otherwise, what can you do? Can you put a spin on it that maybe there is actually something good about it?

**Wojcicki**: Yeah, the FDA is a higher standard than CLIA.

**Gitschier**: In what sense?

**Wojcicki**: In *every* sense. It is a lot more work to get an FDA clearance than CLIA: the amount of validation, the amount of user testing.

**Gitschier**: All right. Let’s talk a little bit about your history leading up to the formation of 23andMe. I know you graduated from Yale in 1996, and somewhere in there you did some actual bench work.

**Wojcicki**: Yeah, I worked with Sid Altman at Yale in 1995, and then I spent the summers of ‘94 and ‘95 at the National Cancer Institute. Then I worked at [the] Weizmann Institute in Israel in ‘95.

**Gitschier**: But when you graduated you went to work on Wall Street.

**Wojcicki**: Yes. After college, I moved home first [to Palo Alto], after driving across the country. I lived at home for a while, and I got this job in New York. I worked for a company called “Investor AB” which is owned by the Wallenberg family in Sweden.

**Gitschier**: And what attracted you to that path?

**Wojcicki**: I had always found biotech stocks fascinating. I loved the biotech space, but I didn’t know that you could actually get paid to research it.

**Gitschier**: Oh, so you were a researcher?

**Wojcicki**: I was a research analyst on Wall Street. People always call me an investment banker. I was not an investment banker. My first project was xenotransplantation. I spent eight months talking to all the experts in the world on xenotransplantation.

**Gitschier**: That sounds like a great job!

**Wojcicki**: It was an amazing job!

**Gitschier**: How could you not learn everything? I think I would like to do that!

**Wojcicki**: There is not a lot on xenotransplantation. If you spend eight months, you get to know the companies, the management teams, the state of the science, all the scientists. You look at it very holistically.

Like today, if I were going to study this problem, I’d look at gene therapy, and I’d want to understand CRISPR/Cas9. I’d want to understand all the companies and the researchers, and I’d want a ten-year vision. Are there companies that fit into that vision that we should invest in, should we start one, or is there something that we own today that we should sell?

**Gitschier**: What companies did Investor AB invest in?

**Wojcicki**: They are the largest shareholder of AstraZeneca, and they own a huge chunk of a company called Gambro which is a dialysis company, which is why we got interested in transplantation. They also owned a bunch in electronics such as Ericsson. It was the first taste I had of the convergence of health and IT [information technology].

**Gitschier**: How long were you at Investor AB?

**Wojcicki**: Until 1998 or 1999. Then I went to a hedge fund, where I was also a research analyst.

**Gitschier**: And when did you leave the hedge fund world?

**Wojcicki**: I took a break in 2000 for about a year. I went through Siberia. I started in Moscow and ended in Beijing.

**Gitschier**: Why Siberia?

**Wojcicki**: My grandmother is from there. I just wanted to go somewhere where there was no Wall Street and no…

**Gitschier**: …internet?

**Wojcicki**: Yeah. There was!

**Gitschier**: Did you go to Mongolia?

**Wojcicki**: I did. Mongolia, Tibet, and Nepal. It was really fun.

And then I went back to the hedge fund world. But part of the purpose of taking off that year was I thought I wanted to go to med school, and so I took the MCATS, which I loved. I was volunteering 40 hours a week at San Francisco General Hospital in the Emergency Department.

**Gitschier**: But you chose not to go to medical school.

**Wojcicki**: Oh, it’s still in the back of my mind! The goal was that I could work one more year in the hedge fund and then I could pay for my medical school bills.

**Gitschier**: Oh, I understand that. Did you actually apply to med school?

**Wojcicki**: No. After Siberia, for the first two years, I was 50/50 in New York and in San Francisco. I flew to New York in the morning of 9/11, and that was my first week back on Wall Street. I got to my hotel at 3 a.m. Then I was on the top floor of the Citibank building at 8 a.m.

The last couple [of] years in the hedge fund world, I wasn’t terribly happy with investing. There was a time period when pharma wasn’t doing a lot of innovative research. It was just about reformulating drugs or maximizing hospital payments, and that wasn’t terribly interesting to me. That was also when I was at San Francisco General.

I started to see this disconnect between what I’m investing in and the people who are actually consuming health care. Nobody loves health care. To me, it was this really sad disconnect between all the people who try to make money off of it, versus the people who need it. I was relatively unhappy, I’d say, for a couple years.

Then I was at a Wall Street event in San Francisco and I met Markus Stoffel, who was a geneticist at Rockefeller. We sat in the back of the room and discussed the utter lack of creativity and just how challenging drug development was. He was the first person I talked to about creating this massive dataset of all this information.

Also, my father is a particle physicist, and he used to look through clinical trials with me. And he’d be like, this is a joke, these statistics don’t make sense, these numbers are so small. These clinical trials, this is what we base our medicine on?

**Gitschier**: One thing that amuses me in the medical field right now is this bandwagon of “evidence-based” medicine.

**Wojcicki**: I know.

**Gitschier**: Excuse me, you mean you’re giving me this pill and there is no evidence that it works?

**Wojcicki**: But often…not! It’s part of how I feel there should be a truth with the consumer.

The last investment area that I was really researching was the genetics space. Affymetrix was one of the companies I had known from 1996. I’d meet these people who said, “It’s amazing—the genetics landscape is so exciting and everything is changing.” I realized that it was cheap enough that you could actually empower people to get access to their own information. And there was such a crowdsourcing movement. It was the beginning of Web 2.0. The idea was to actually let people have access to their genomes and enable them to learn about science. Then I met Linda [Avey], who was at Affymetrix, and they had this idea about a consumer testing company, so the two ideas really merged.

**Gitschier**: Tell me a little more about that meeting.

**Wojcicki**: Linda was working on a Michael J. Fox project on Parkinson’s. She had met Sergey, and I had come to the meeting. That was the first time we met.

**Gitschier**: What date was the company founded?

**Wojcicki**: It was April 27th of 2006. We decided to start the company on Valentine’s Day of 2006. And I must have met Linda around November of 2005. I would have to check my email.

**Gitschier**: Oh, you save your emails.

**Wojcicki**: Everything, of course.

**Gitschier**: Getting back to the FDA, now that you can’t deliver medical information for the time being [except for on Bloom Syndrome], we’ve got all these third-party interpreters out there—DIY Genomics, Promethease, Athletigen, Interpretome, to name just a few—who take the data from people who have been genotyped by 23andMe and deliver disease-risk information back to them. You know there is an irony here. 23andMe is prohibited from this final step, but for five bucks you can send your results from 23andMe and make an end-run around the FDA.

**Wojcicki**: Actually, the FDA has commented that any software that interprets medical information from the human genome can also be considered a medical device and be subject to regulatory review.

**Gitschier**: Ah, interesting. We’ll have to see how that all plays out.

**Wojcicki**: We have an API [Application Program Interface], a standard tool in the tech world to enable people to pull down their data. That way, Promethease and all these others can build tools on top of the infrastructure that we have. We try to encourage use for art or music or other things that we just aren’t doing. It’s your data—you can do all kinds of things—explore it! We want to foster that industry of others bringing your data to life in ways we don’t, and we’ve had other research groups use data for medical information [directly from our clients].

**Gitschier**: Speaking of data, I logged into my own data prior to our interview to see if there is anything new.

**Wojcicki**: There is new stuff added only if it is not health-related since November 22, 2013 when the FDA handed down the cease-and-desist order.

**Gitschier**: How can you tell what counts as health-related?

**Wojcicki**: We have a very active dialogue.

**Gitschier**: OK, so whether or not I sneeze when I see sunlight is not regulated by the FDA.

**Wojcicki**: It is not regulated by the FDA so long as I am not telling you that sneezing when you look at sunlight is associated with ovarian cancer.

**Gitschier**: Which you are not going to tell me since there is no such association. Just to be clear.

**Wojcicki**: These are all things that we have dug into!

**Gitschier**: I noticed, of course, that you just recruited two prominent guys from Genentech—Richard Scheller and Robert Gentleman.

**Wojcicki**: They are both awesome.

**Gitschier**: So, what’s the plan there?

**Wojcicki**: We want to develop drugs.

**Gitschier**: When you say “develop drugs,” do you mean literally put them in a bottle? Do clinical trials?

**Wojcicki**: We have all these consumers who came to us because they want to learn about themselves, but they also participate in research because they want to extract meaning out of the genome.

The idea of Richard and his team is to really look at the database and ask can we find insights that would lead to drug discovery that would have a better success rate than the current industry standard, which is that nine out of ten fail. Are we going to stop at preclinical, are we going to stop at Phase I or Phase II? We have no idea until we know what we are going to pursue. We don’t have to make any of those decisions until later down the road, if we found something that is so compelling. We can decide: do we want to partner, or do we want to pursue it on our own?

**Gitschier**: Nice new building, by the way. You just outgrew the old one?

**Wojcicki**: We more than outgrew it—we were in trailers. This is our fifth place. We started in 2006 in a coffee shop where we used to meet. The second place was an apartment on Alma Street. Our third place was a sublet from SETI—the search for extraterrestrial life.

**Gitschier**: Oh yeah? I love that!

**Wojcicki**: We got a lot of their mail. A lot of new age stuff. The fourth site was our first real office space, where we had a glass conference room! That was fancy. Number 5 was on Shorebird Way. We were there for a long time—four years. We got kicked out, because they wanted the building back.

**Gitschier**: Well, this is very lovely!

**Wojcicki**: This is a lot more expensive. It’s lovely for a reason.

**Gitschier**: I’ve heard you talk online about fostering work/life balance for your employees, and my question is, how do you deal with your own work/life balance?

**Wojcicki**: [Laughter] To me, the reality is that you kind of work all the time, but it’s not black and white where you work 9 to 5 and then go home and I don’t work. It gets integrated quite a bit.

The one area that I really sacrifice on, which I would say everyone here realizes, is that I never get dressed nicely. I always bike to work. So I wear the same thing every day. [Laughter] For me, the idea of spending time on personal appearance is like…just not worth the time.

Two, I definitely set boundaries for when I get to see the kids. I try to spend at least one day a week where I volunteer in the school, and I encourage others to do that too. It makes a world of difference, and then it doesn’t really matter if I say, “I can’t see you for two days” because I came to school that one day.

People here at the office definitely take that children time—when you pick up your kids, you’re with your family for dinner, you put the kids to bed—and then people are very active online afterwards. There is a prolific amount of email after 10 p.m.

Part of why we chose this location—and we paid more for it—is that if I can make people’s lives easier by being on the train line, then it is worth the money. Even things like having the food brought in, and we’re dog friendly. There is a big dog area; I don’t know where it is, but there are a lot of dogs! But it just makes it better. It is just part of the culture.

**Gitschier**: So this comes to the heart of the matter for me. What is it that really drives you? Because this is obviously the love-of-your-life kind of thing. And then the follow-up question is, what is it about your particular background or skillset that you think makes you well-suited to this?

I’m thinking about this because when I have considered start-ups and talked to venture capital people, they always comment, “Well after five years, we’ll sell this.” And I’m thinking, why should I spend five years of my life if you want to sell this off to somebody else? So, you’ve obviously made it through the five-year point, and you are still here!

**Wojcicki**: That was part of the capital structure. Because I come from Wall Street, I understood that for people who provide capital, their incentives are different than entrepreneurs’. Part of the reason that Sergey was supportive and that we took more money from individuals and from Sergey was that we wanted to have more of that structure, where we make decisions that are in the best interests of the company, not necessarily in the best interests of getting your capital out. That is why we have not gone public.

Also, I think that for our mission, it’s really important that people trust the company. That was part of the reason that I wanted to make sure that it was owned more by me. Control is really important to me. And obviously we have significant shareholders as well, and all of our employees are shareholders.

**Gitschier**: So, back to this issue of …

**Wojcicki**: Why do I do this? Three things. One, when I worked in the lab with Sid Altman, I loved that moment when you get that answer. It’s so exciting! And in some ways, it’s kind of sad because you spend so much time trying to get to a discovery and then you get it, and it’s so—ugh—the experiment didn’t work, or it’s not what you thought, or you have to do it again. But it’s exciting sometimes.

Part of what is exciting here is that we’ve really accelerated the pace of discovery. Like we just did user group testing the other day, and it was interesting because everyone has their questions, like should I be drinking coffee?

**Gitschier**: Absolutely you should be drinking coffee!

**Wojcicki**: I know—my mom even said coffee is good. But the question is whether coffee is right for *me*. The beauty of the internet today is that you can get all these answers, but there is nothing that is about *you*.

One thing that I really learned from Wall Street, which I think was unique compared to everyone with an MD or PhD, is that I really understand how it works. In some sense, I know enough about it to understand that I have no idea how it works! It so disjointed! Understanding who is actually the decision maker is super complicated!

But the one thing I know for sure is that you, the customer is never the decision maker. You aren’t in charge of your own health in any capacity because you don’t pay the bills. You don’t decide whether your insurance company is going to pay. You don’t decide whether or not you’re going to get a certain type of treatment.

Consider Nordstrom: Nordstrom has a great customer service policy; they are really nice to you because *you* are the customer. But you are no one’s customer in health care. No one really cares about customer satisfaction. The only thing that they do—in a kind of disgusting way—is that they lock you up. The hospitals want you to come back, but they do that by trapping your medical records there.

**Gitschier**: And trapping your insurance.

**Wojcicki**: Right. It’s not that you decided, “Oh, this is so great.” It’s just so hard to move!

Health is the ultimate equalizer. It doesn’t care how much money you have, your race, your gender. When we are really sick, we all want the same thing. I get irrationally crazy when I see people who are really sick and people taking advantage of them. The health care system makes decisions on your care because it’s in the best interest of the hospital, or best interest of the insurance company, or the best interest of the pharmacy manager.

Part of this also comes from my mother. You know this story? My mother had a little brother who died from eating a bottle of aspirin when he was 18 months old. My mother has always been irrationally aggressive in health care. And I see how people don’t have enough advocates. I’m just super passionate about that moment in time when you should really be cared for!

So: One, I love that moment of discovery; I love accelerating discovery. Two, by understanding the health care system, I have had an advantage of recognizing how complicated it is. Three, I have an extraordinary passion for the individual and the consumer having a say in his or her own health care. And when we think about what is going to change health care, consumer involvement is a major part of that. “You” becoming more and more the customer changes how everyone in health care treats you.

**Gitschier**: So, you are squarely in middle age now, I would say. Are there things that you can look back on and say, wow—these were the best things that happened to me in my life—most exciting, joyful, whatever term you want to use, and then the opposite, too.

**Wojcicki**: Two things. First, when I quit my job in 2000, it was the top of the market, and it was one of the best things I have ever done. When you are in the thick of it you think, “Oh I can’t, my career has this going on.” I think it is really important to. The reality is that between 20 and 30, everyone gives you that discount, so take advantage of it! Do all the crazy things, because you are only 27. The freedom of that year, taking off, that is the top hit!

Second, when I graduated from college without a job. Like my sister [Susan Wojcicki], who is the CEO of YouTube: in her first job, as a temp, she was working with the Palo Alto Sanitation Company. It’s one of those things where there is nothing that is beyond you. During my first summer after college, I temped at all kinds of offices: two weeks babysitting, two weeks at Montgomery Security, two weeks in a law office, just to see what it was like.

**Gitschier**: What did you learn about yourself?

**Wojcicki**: One thing is that I won’t do things that are just for money. That one year I worked for somebody that I absolutely loathed, and I only did it because I knew I would make money. Every day of that year was miserable, and I shouldn’t have done it.

I think one of the best things that my parents taught me is how to live with no money. When I first moved to New York, I made $40,000 and I saved $1,000 a month. I had a food budget of $15. It’s purely just discipline.

**Gitschier**: Your parents both had incomes, so how did they teach you that?

**Wojcicki**: They were super frugal and super focused on the importance of saving money. And also questioning what you really need. My mom always said you need a pair of shoes, and you need one pair of underwear, because you always wash it, and maybe two shirts. When I did the Siberia trip I had literally two pairs of underwear. You don’t need anything. You can always find food.

I have somewhat of a luxury because I’m educated. I always feel that I can always find a job. I always have ideas, and I can always do something. I don’t know what, but I can guarantee you that I can make money in some way.

**Gitschier**: I’m not sure everybody has that same feeling.

**Wojcicki**: But I don’t know why! When you look in the developing world, where there is not an option to go and get a job at Google or GE, you have to be thrifty and figure it out. You have to be creative. That’s what my mom made us do. She made us always come up with different ideas. If you want something or want to do something, then you figure it out.

**Gitschier**: It sounds like your parents were pretty amazing, and I know your whole family all lives right around here. What’s is like when you all get together for dinner?

**Wojcicki**: My sister Susan came over on Sunday, and I was like, “Why are you wearing my shirt? Why are you stealing my clothes?”

**Gitschier**: How can she steal your clothes?

**Wojcicki**: She comes to my house, she goes upstairs, and she steals my clothes!

**Gitschier**: Still? I have to tell you, I saw a picture of her and one of you and I think you are wearing the same dress.

**Wojcicki**: Well, I now buy stuff for all three of us! I’m the only one who buys clothes.

**Gitschier**: But you just told me…

**Wojcicki**: Occasionally I have to dress up.

**Gitschier**: Yeah, I’d say so.

**Wojcicki**: It is more fun for me. Like my sister Susan at YouTube, I say, “You’re like a media celebrity, I’ve got to buy you stuff!” And my other sister, she is more academic, and it is fun to torture her with, “Hey, look at what I bought you!”

I see my family all the time, and I see all my neighbors all the time, and we still regularly do events. My kids are on the same swim team I swam on at Stanford, as are the kids of all my friends whom I swam with, and then the coach was trained by my coach, and it was one of my mom’s students.

**Gitschier**: Your mother used the words “Tikkun Olam.” World repair.

**Wojcicki**: My mother used this?

**Gitschier**: Yeah. It’s Hebrew—to heal the world. She was talking about what she believes in and perhaps instilled in her three daughters. I suppose you are addressing how 23andMe is making the world better.

**Wojcicki**: The mission is about trying to change health care through empowering you. I think there is a whole cascade of effects that will come if we can actually empower you to have more ownership. My hypothesis is that people are not proactive about their health because they don’t feel they are empowered. If you are treated like a child all day—just look at the communist system—you are not really inspired to take ownership. I want to change that.

